# Tissue Doppler echocardiographic quantification. Comparison to coronary angiography results in Acute Coronary Syndrome patients

**DOI:** 10.1186/1476-7120-3-10

**Published:** 2005-04-08

**Authors:** Erwan Donal, Pascale Raud-Raynier, Damien Coisne, Joseph Allal, Daniel Herpin

**Affiliations:** 1 Department of Cardiology, University Hospital La Miletrie, 86021 POITIERS – France

**Keywords:** Acute myocardial infarction, Doppler Tissue Imaging, Echocardiography

## Abstract

**Background:**

Multiples indices have been described using tissue Doppler imaging (DTI) capabilities. The aim of this study was to assess the capability of one or several regional DTI parameters in separating control from ischemic myocardium.

**Methods:**

Twenty-eight patients with acute myocardial infarction were imaged within 24-hour following an emergent coronary angioplasty. Seventeen controls without any coronary artery or myocardial disease were also explored. Global and regional left ventricular functions were assessed. High frame rate color DTI cineloop recordings were made in apical 4 and 2-chamber for subsequent analysis. Peak velocity during isovolumic contraction time (IVC), ejection time, isovolumic relaxation (IVR) and filling time were measured at the mitral annulus and the basal, mid and apical segments of each of the walls studied as well as peak systolic displacement and peak of strain.

**Results:**

DTI-analysis enabled us to discriminate between the 3 populations (controls, inferior and anterior AMI). Even in non-ischemic segments, velocities and displacements were reduced in the 2 AMI populations. Peak systolic displacement was the best parameter to discriminate controls from AMI groups (wall by wall, p was systematically < 0.01). The combination IVC + and IVR< 1 discriminated ischemic from non-ischemic segments with 82% sensitivity and 85% specificity.

**Conclusion:**

DTI-analysis appears to be valuable in ischemic heart disease assessment. Its clinical impact remains to be established. However this simple index might really help in intensive care unit routine practice.

## Introduction

Tissue Doppler echocardiography (DTI) has been introduced as a new method to quantify regional myocardial function. It provides an analysis of myocardial velocities and regional myocardial displacement, deformation and rate of deformation [1-5]. In routine practice the diagnosis of an acute coronary syndrome is based on guidelines using clinical, electrocardiographic and biological patterns [6]. Nevertheless to improve speed, pertinence and efficiency of the diagnosis in many patients, a subjective interpretation of myocardial regional motion and thickness is frequently performed in the intensive care unit (ICU), therefore not necessarily in the best conditions. The, wall motion score assessing regional contractility has been validated. Reproducibility of this method is high for expert observer but it requires dedicated training and remains subjective. Thus, DTI may offer an alternative quantitative technique that can be used in routine practice. Several parameters have been described in experimental settings [7-11]. We therefore sought, to look at most of these previously proposed quantitative parameters in a clinical setting to assess their relevance compared to coronary angiographic findings.

## Methods

This study was a single-center observational study. Informed consent was obtained prior to each coronary angiography. The study was designed in compliance with the ethic principles of our institution.

A series of consecutive acute myocardial infarction (AMI) patients treated by primary percutaneous coronary angioplasty (PTCA) of the left anterior descending coronary artery (15 patients, 66.5 ± 17 years of age) or the right coronary artery (13 patients, 54 ± 13 years of age) were prospectively analyzed. The TIMI 3 flow was obtained in all patients. The exclusion criteria were: AMI related to circonflex artery (this coronary artery might be responsible of too variable size and localization to constitute an homogeneous group), any other coronary artery stenosis than the culprit lesion, left bundle branch block, pace maker, significant valve disease, atrial fibrillation, dilated or hypertrophied cardiomyopathy and cases in which more than twelve hours had elapsed between the beginnings of the chest pain to the coronary angiography. We did not included patient with a circonflex artery lesion in this study because the myocardial territory vascularized by this artery might be too much different from one patient to another one.

The control group consisted of 17 patients without any known myocardial disease who had been referred to the catheterism laboratory for chest pain. Each of these patients had a normal coronary angiography and normal conventional trans-thoracic echocardiography.

For the 2 AMI groups, the echocardiographic exam was performed in the 24 hours following the admission and the PTCA. Each of these patients had no other known pathology likely to impact on myocardial function and the treated artery (right coronary and left anterior descending coronary artery) was the only abnormal artery on the coronary angiography. The angiographic result of each PTCA performed was satisfactory and a TIMI 3 flow was recorded in all patients at the end of the procedure.

The same investigator performed all the echocardiographic recordings and analysis. A Vivid Five ultrasound scanner (GE VingMed, Milwaukee, Wisconsin) with a 2.5 Mhz phased array transducer was used.

The patients were imaged in the left lateral decubitus position in the ICU. Standard 2-dimensional, M-mode, pulsed and color Doppler were saved on digital files (Echopac – GEVingMed, Milwaukee, Wisconsin). In apical four and two-chamber, color DTI Cineloops of 3 cardiac cycles (from a gating EKG) were recorded with a frame rate between 80 and 115 frames/s depending on the sector width. The image was adjusted to ensure a parallel alignment of the sampling window with the myocardial wall studied. The pulse repetition frequency was between 500 Hz and 1 KHz, resulting in an aliasing velocity about 16 to 32 cm/s. Three cardiac beats were digitized and stored on a magneto-optical disk for further analysis on Echopac (GE VingMed, Milwaukee, Wisconsin).

### Data processing and analysis

**Figure 1:**
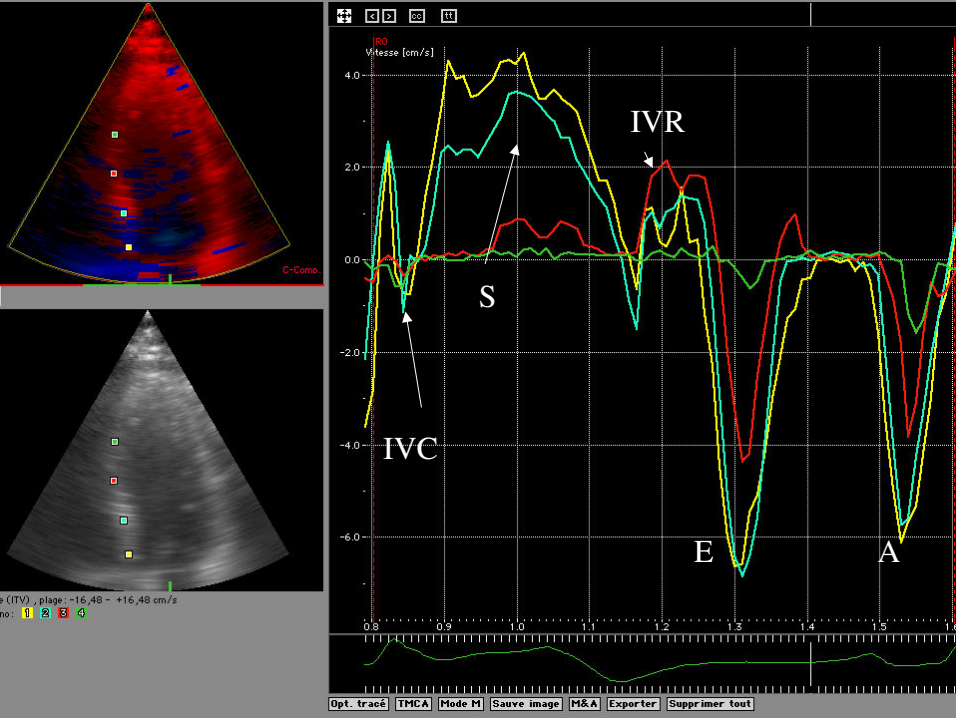
Example of a DTI curve analysis. It was manually positioned a region of interest (ROI) at the level of the mitral annulus and three others in the 3 segments of each wall (following the ASE left ventricular segmentation) IVC: peak of velocity recorded at the isivolumic contraction time S: peak of velocity recorded in systole IVR: peak velocity recorded in isvolumic relaxation time E: peak velocity in early diastole A: peak velocity in end-diastole

**Figure 2:**
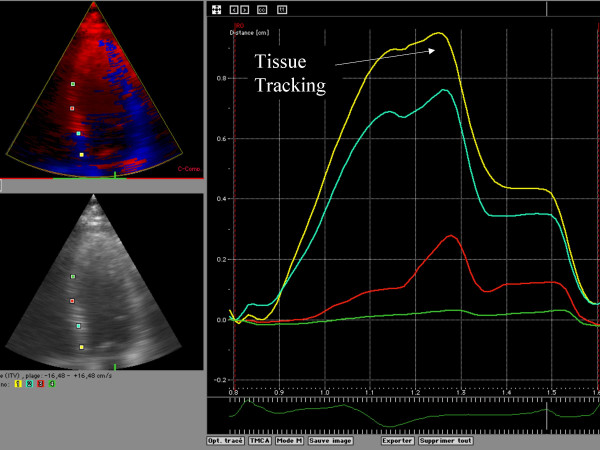
Example of Tissue tracking curves displayed on Echopac. It was there easy to record the peak (S) of systolic displacement of the same 4 ROI described in figure 1.

**Figure 3:**
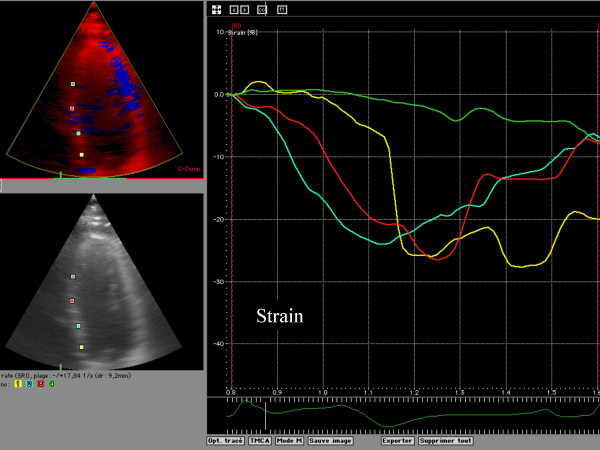
Example of Strain analysis; It was there possible to record the degree of systolic shortening or lengthening of the 4 studied ROI.

Myocardial Doppler velocity profile signals were reconstructed offline from the DTI color images. Curve analysis was performed on an averaged analysis of the three recorded cardiac beats. A regional analysis was then performed, positioning an index at the mitral annulus, basal, mid and apical segments of the septal, lateral, anterior and inferior left ventricle myocardial wall (figure 1). The systolic (S); early diastolic (E), late diastolic (A) and also the isovolumic contraction and relaxation peaks were recorded. Tissue tracking systolic peak displacement was recorded for each of the 4 indices placed on each wall (figure 2). The strain rate analysis was considered, but we chose to not report any results. The signal to noise ratio was not acceptable. Frequently, it was challenging to distinguish peaks in systole and diastole. Nevertheless, we considered the systolic peak of strain and the delay of apparition of that peak of contraction for the analysis (figure 3).

To evaluate left ventricular filling pressures, the ratio mitral inflow E-wave peak velocity / early diastolic velocity Ea of the mitral annulus (septal part) was calculated. Ea being the early diastolic pulsed DTI peak velocity recorded in pulsed DTI [12].

### Statistics

Before comparison, the 3 grouped datasets were tested for normal distribution and equality of standard deviation (SD). Normally distributed data are given as mean and SD, and were compared using a parametric analysis of variance (ANOVA, completed by a Scheffe post-hoc analysis). Tests resulting in p values below 0.05 are considered statistically significant. Receiver operating characteristics and logistic regression analysis were used to find and optimize cut-off values of the most relevant parameters for distinguishing normally vascularized myocardial segments and myocardial segments just after revascularization by emergency coronary angioplasty for signs of AMI.

To assess the intra and inter-observer variability, each echographic parameter was determined again by the original observer and an independent observer in 10 patients from the 3 populations.

## Results

### Clinical and echocardiographic characteristics

Table 1 (additional file 1) summarizes the clinical characteristics of the three populations. Pharmacologic therapy is identical in the two AMI populations. Each patient was receiving Metoprolol Succinate. The mean dosage was 100 mg per day or Atenolol 50 to 100 mg per day.

In control subjects: The isovolumic contraction (IVC) was a positive wave. The systolic (S) wave was positive too. The isovolumic relaxation time motion (IVR) was negative as were E (early relaxation motion) and A (late diastolic motion).

**Figure 4:**
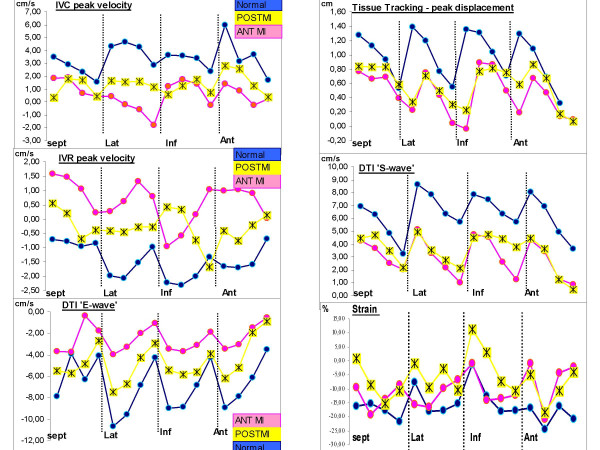
Representation of IVR (isovolumic relaxation), IVC (isovolumic contraction) peak velocities, early diastolic tissue Doppler velocity (E), systolic tissue Doppler velocity (S), systolic displacement (tissue tracking) and strain in the three segments of each myocardial walls analyzed in the anterior (ANT) myocardial infarction population, in the inferior myocardial infarction one (POST) as in the control group (normal).

Figure 4 shows the mean values of the most pertinent DTI-derived parameters in each wall for the 4 points that were studied (annulus, base, mid-segment and apex). This figure 4 illustrates that even in non-ischemic segments from the 2 AMI populations (the inferior and the anterior one), velocities and displacements were significantly altered in comparison to the control population. Potentially linked to these findings, estimated left ventricular filling pressures were significantly higher in the two AMI groups:

E/Ea = 18 ± 12 (cm/s) in the Anterior AMI group;

E/Ea = 16 ± 10 (cm/s) in the Posterior AMI group;

E/Ea = 8 ± 2 (cm/s) in the control group.

### Analysis of the differences between each studied parameters using variance analysis

The E and the S waves of the DTI appeared relevant to discriminate the myocardial properties of the three studied populations, especially in the basal and mid segments of each wall (p-value always ≤ 0.03) (additional file - table 2).

The IVR peak velocities were highly discriminative when the control subjects were compared to each of the two AMI populations, considering the values observed in the basal and mid segments. P-value was <0.005 for each wall except the inferior wall for which IVR did not discriminate either of the two AMI populations in the mid segment and p-values were only 0.02 in the basal segment.

The peak of systolic displacement analyzed in tissue tracking mode was extremely relevant, always discriminating the three myocardial status with p-values < 0.003.

There was no substantial change in the peak IVC, the delay of S-wave and the A-wave between the control population and each of the two AMI populations. The systolic peak of strain was not discriminative enough to distinguish normal from abnormal myocardium (p = ns).

### To define the best parameter or the best combination of parameters against the coronary angiography results, we performed a logistic regression analysis

To discriminate the control subject from the pathologic subjects, the peak of systolic displacement measured by tissue tracking appeared to be the best parameter at the annulus and the basal segment level (β = -14.27, p = 0.0018 ; β = -10.36, p = 0.005; β = -17.75, p = 0.01; β = -12.09, p = 0.01 in the septal, lateral, inferior and anterior wall respectively). Results were less clinically relevant in the mid and apical segments. The multivariate analysis did not provide one parameter, but three different ones form one segment to another.

To discriminate anterior from inferior AMI, peak E was the best parameter at the mitral annulus and peak IVR was the best in the basal and mid-segments of each left ventricular walls (β = -1.14, p = 0.011; β = 0.94, p = 0.014; β = -1.74, p = 0.025; β = 1.97, p = 0.059 in the septal, lateral, inferior and anterior walls respectively).

To complete the determination of the most clinically relevant parameters to distinguish normal from recently revascularized segments, it was performed a ROC curve analysis. At this point, it was chosen to implement a qualitative approach based on our experience of DTI signal analysis. Thus, it was added in the analysis the parameter compiling IVC peak > 0 and IVR < 1. The qualitative parameter was added because of the observation that we did during the analysis of curves and quantitative parameters. This "IVC peak > 0 and a IVR peak < 1" enabled to exclude coronary artery disease for the studied segment with 82% sensitivity and 85% specificity. The predictive positive value was 68% and negative predictive value 92%.

For IVR peak velocity, the area under the curve was 0.73; sensitivity was 72% and specificity 69% for a cut-off value of less than 0.12 cm/s to predict the absence of coronary artery lesion.

For the peak VTI (tissue tracking), a cut-off value of less than 0.78 cm offered 76% sensitivity for 51% specificity in predicting the presence of a coronary lesion treated by PTCA in the last 24 hours. The area under the curve was 0.68.

**Intra and inter observer variabilities **are shown on table 3 (additional file 3). Average variability over each wall and each segment are shown. No data are reported for the strain rate in the table. Relative strain rate variability was around 10% in the basal segments and reached 50% of variability in some of the apical ones.

## Discussion

In the current study, the main findings were: (1) in an AMI population, myocardial velocities and displacement were found to be deteriorated in the AMI territory but were also deteriorated in the non-ischemic left ventricular myocardial walls. (2) isovolumic relaxation peak velocity seemed to be relevant taken alone and even more in conjunction with isovolumic contraction peak velocity. (3) Peak systolic displacement provided by tissue tracking was easy to obtain and relevant to detect the AMI population and/or AMI territories 12 hours after acute revascularization of an anterior or inferior AMI. (4) Strain and strain rate analysis appeared to be less relevant than expected from a physio-pathologic state points and taking into account studies done under stringent experimental conditions.

A qualitative approach based on visual assessment of myocardial wall motion has well-documented limitations and even with eyes of an expert may fail to identify subtle ischemia induced changes in regional myocardial mechanics [13]. In our practice a hypokinesia was observed in one or two segments of the territory of the treated coronary artery. Nevertheless, the hyperkinesia frequently observed in adjacent segments often altered our capacity to precisely define what could be called the "region at risk". This limit and the observer dependency were our initial motivation in studying DTI parameters. The curve analysis and especially the qualitative analysis of isovolumic relaxation and contraction peak velocity answer to our initial goal. It becomes possible to record images and to discuss between physicians with the help of this curves analysis. Furthermore we showed that no AMI segments were having also abnormal values of DTI parameters compared to the control group myocardial segment. Age is probably not the explanation to this difference, as evoked by the E/Ea ratio; left ventricular end-diastolic pressure may have an impact on regional myocardial function even in non AMI segments [14]. Most studies dealing until now, with DTI abilities have been conducted in stringent experimental settings; also most of them postulated that one specific parameter or one specific DTI – derived post-treatment was sufficient to distinguish stunned or ischemic myocardial segments from others, normally vascularized or scarred [15-19]. The most recent studies have emphasized the great capability of deformation indices derived from strain and strain rate analysis. The accuracy of such ultrasonic strain measurements has been validated in a comparison with sonomicrometry in experimental ischemic models, and ranges of normal values have been recently established in healthy volunteers [20,21]. The main advantages of these myocardial regional deformation parameters comes from the fact that they are less affected by global cardiac motion and segmental tethering than velocity measurements [22-25]. Nevertheless, they remain sensitive to noise and reproducibility does not reach sufficient robustness. An expertise might be request to perform the interpretation of strain and even more, strain rate curve analysis and one has to keep in mind that our study was performed in the intensive care unit characterized by its monitoring of EKG, pressure, SaO_2_. New softwares, dealing with higher frame-rate images might offer new opportunities to use strain rate, but it remains in our mind a research tool difficult to use in the clinical routine [25,26].

Tissue tracking and, thus the measurement of peak systolic regional displacement is a more recently available post-treatment [27,28]. The obtained curves are much easier to interpret. This might encourage the performance of this measurement in routine practice to discriminate AMI myocardial walls even if the tethering effect, due to the fact that one analyzes velocities (integrated over time), might decrease its theoretical sensitivity. This signal processing method has several advantages: it can be used for off-line measurements and it could also be used on-line to display a color map (parametric imaging) presentation of the degree of systolic longitudinal displacement of each studied myocardial segment. Tissue tracking peak systolic regional displacement has been validated in ischemic heart disease and in stress echocardiography interpretation [29,30]. In our opinion it needs no specific-training, and it has been demonstrated to be relevant especially at the basal part of the left ventricle [29].

Isovolumic peaks, especially isovolumic relaxation peak (IVR) velocity, have been reported in the past and appeared to be relevant [11,31]. In this present study, we underscored the advantage of looking at the positivity or the negativity of the relaxation and contraction isovolumic peaks, without any precise measurement. *Pislaru et al *reported, in an experimental model, that the presence of a positive IVC velocity wave in the ischemic wall was a marker of a less severe ischemic insult, while its absence identified a more severe ischemia [31]. IVC tissue velocities were more sensitive in terms of the trans-mural extension of necrosis than other systolic and diastolic regional velocity parameters. They also reported that the systolic DTI wave was of little value to discriminate ischemic from non ischemic myocardial segment. *Edvarsen et al *reported previously IVC and IVR velocity waves relevance in differentiating ischemic from non-ischemic myocardium [11]. These isovolumic indices take advantage of being fewer loads dependent and perhaps also easier to assess than strain and strain rate signals.

### Limitations

Our control population is not matched in age. Ethic consideration prevent to include patient without any indication for a coronary angiography. We were nevertheless able to compare ischemic segments from non-ischemic ones in the two AMI groups. We do not reported the wall motion score analysis because it was not our purpose. Our daily practice provided us doubts in the ability of wall motion score performed within the first 24-hour in the intensive care unit to really discriminate the "region at risk".

## Supplementary Material

Additional File 1 Epidemiological and general echocardiography characteristics of the three studies group and comparison of them. (Ant MI: anterior myocardial infarction; Inf MI: inferior myocardial infarction; LV: left ventricle; IVRT: isovolumic relaxation time; PVF: pulmonary vein flow; TEI: Tei index or Myocardial performance index; E: peak of the velocity recorded in early diastole at the mitral inflow; Ea: peak of velocity recorded in early diastole at the level of the septal part of the mitral annulus in pulsed DTI; E-wave DT: deceleration time of the E-wave). The differencies between the three group by the one-way ANOVA was highly significant: ** = p < 0.001 or significant:* = p < 0.05. It is also display the differences group per group when the ANOVA showed a significant difference (Scheffe post-hoc analysis). Click here for file
