# Supplementary material for: Tissue Doppler echocardiographic quantification. Comparison to coronary angiography results in Acute Coronary Syndrome patients
Source: Cardiovasc Ultrasound. 2005 Apr 8;3:10. doi: 10.1186/1476-7120-3-10 (PMC1084356; doi:10.1186/1476-7120-3-10)
Supplement: Additional File 1 — Epidemiological and general echocardiography characteristics of the three studies group and comparison of them. (Ant MI: anterior myocardial infarction; Inf MI: inferior myocardial infarction; LV: left ventricle; IVRT: isovolumic relaxation time; PVF: pulmonary vein flow; TEI: Tei index or Myocardial performance index; E: peak of the velocity recorded in early diastole at the mitral inflow; Ea: peak of velocity recorded in early diastole at the level of the septal part of the mitral annulus in pulsed DTI; E-wave DT: deceleration time of the E-wave). The differencies between the three group by the one-way ANOVA was highly significant: ** = p < 0.001 or significant:* = p < 0.05. It is also display the differences group per group when the ANOVA showed a significant difference (Scheffe post-hoc analysis). [file 1476-7120-3-10-S1.doc]

|  | Body area (cm2) | Age | LV End-diastolic diameter (cm) | Indexed LV masse | IVRT | E-wave  (m/s) | E-wave  DT  (msec) | A-wave  (m/s) | E/Ea  (cm/s) | Tei  (msec) | LV outpout | LV ejection fraction |
| --- | --- | --- | --- | --- | --- | --- | --- | --- | --- | --- | --- | --- |
| Ant MI | 1.7±0.2 | 66.5±16.9 | 50.5±6.4 | 144±36 | 89±27* | 0.5±0.1 | 184±71.5 | 0.7±0.3 | 18±1.1 | 0.72±0.24 | 5.0±1.4 | 0.49±0.13 |
| Inf MI | 1.9±0.2 | 53.8±12.7 | 49.6±6.3 | 115±41 | 68±20 | 0.7±0.1 | 140.5±25.2 | 0.6±0.2 | 14±6.0 | 0.67±0.07 | 5.0±1.4 | 0.49±0.13 |
| Control | 1.8±0.2 | 49.0±11.2* | 49.0±4.5 | 113±34 | 71±24 | 0.6±0.1 | 177.4±46.1 | 0.6±0.1 | 8±2.0* | 1.21±0.3* | 5.8±1.6* | 0.62*±0.09 |
| ANOVA | ns | ** | ns | ns | * | ** | ns | ns | ** | ** | ** | ** |

**Table 1:**

Epidemiological and general echocardiography characteristics of the three studies group and comparison of them. (Ant MI: anterior myocardial infarction; Inf MI: inferior myocardial infarction; LV: left ventricle; IVRT: isovolumic relaxation time; PVF: pulmonary vein flow; TEI: Tei index or Myocardial performance index; E: peak of the velocity recorded in early diastole at the mitral inflow; Ea: peak of velocity recorded in early diastole at the level of the septal part of the mitral annulus in pulsed DTI; E-wave DT: deceleration time of the E-wave). The differencies between the three group by the one-way ANOVA was highly significant : ** = p<0.001 or significant:*=p<0.05. It is also display the differences group per group when the ANOVA showed a significant difference (Scheffe post-hoc analysis).

|  | ***DTI S-wave*** |  |  | ***DTI E-wave*** |  |  | ***IVC peak*** |  |  | ***IVR peak*** |  |  | ***Tissue Tracking*** |  |  |
| --- | --- | --- | --- | --- | --- | --- | --- | --- | --- | --- | --- | --- | --- | --- | --- |
|  | **ANT/INF** | **INF/0** | **ANT/0** | ANT/INF | **INF/0** | **ANT/0** | **ANT/INF** | **INF/0** | **ANT/0** | **ANT/INF** | **INF/0** | **ANT/0** | **ANT/INF** | **INF/0** | **ANT/0** |
| **Septum annulus** | 0.68 | <0.001 | <0.001 | 0.01 | 0.002 | <0.01 | 0.48 | 0.006 | 0.03 | 0.19 | 0.02 | <0.001 | 0.4 | <0.001 | <0.001 |
| **Septum base** | 0.03 | 0.001 | <0.001 | 0.01 | 0.01 | <0.001 | 0.8 | 0.06 | 0.09 | 0.006 | 0.16 | <0.001 | 0.1 | 0.003 | <0.001 |
| **Mid-Septum** | 0.02 | 0.001 | <0.001 | 0.03 | 0.02 | <0.001 | 0.12 | 0.1 | 0.001 | 0.002 | 0.3 | <0.001 | 0.1 | 0.002 | <0.001 |
| **Lateral annulus** | 0.78 | 0.002 | <0.001 | <0.001 | 0.001 | <0.001 | 0.35 | 0.03 | 0.001 | 0.42 | 0.005 | <0.001 | 0.1 | 0.009 | <0.001 |
| **Lateral base** | 0.78 | <0.001 | <0.001 | 0.002 | 0.008 | <0.001 | 0.12 | 0.007 | <0.001 | 0.11 | 0.006 | <0.001 | 0.6 | <0.001 | <0.001 |
| **mid lateral** | 0.53 | <0.001 | <0.001 | 0.03 | 0.01 | <0.001 | 0.17 | 0.003 | <0.001 | 0.004 | 0.03 | <0.001 | 0.007 | <0.001 | <0.001 |
| **Inferior annulus** | 0.66 | <0.001 | <0.001 | 0.02 | <0.001 | <0.001 | 0.64 | 0.001 | 0.04 | 0.004 | <0.001 | <0.001 | 0.02 | <0.001 | <0.001 |
| **Inferior base** | 0.70 | <0.001 | <0.001 | 0.03 | 0.002 | <0.001 | 0.39 | 0.01 | 0.01 | 0.02 | <0.001 | <0.001 | 0.18 | 0.002 | <0.001 |
| **Mid-Inferior** | 0.001 | 0.003 | <0.001 | 0.001 | 0.2 | <0.001 | 0.9 | 0.01 | <0.001 | 0.002 | <0.001 | <0.001 | 0.01 | <0.001 | <0.001 |
| **Anterior annulus** | 0.83 | <0.001 | <0.001 | 0.01 | 0.009 | <0.001 | 0.1 | <0.001 | <0.001 | 0.003 | 0.008 | <0.001 | <0.001 | 0.002 | <0.001 |
| **Anterior base** | 0.91 | <0.001 | <0.001 | 0.02 | <0.001 | <0.001 | 0.9 | 0.2 | 0.16 | <0.001 | 0.04 | <0.001 | <0.001 | <0.001 | <0.001 |
| **Mid-Anterior** | 0.79 | <0.001 | <0.001 | 0.5 | <0.001 | <0.001 | 0.03 | <0.001 | <0.001 | 0.01 | 0.04 | <0.001 | <0.001 | <0.001 | <0.001 |

**Table 2:** Analysis of variance to compare control (0) and anterior acute myocardial infarction (ANT) and inferior myocardial infarction (INF) populations. It is displayed the p-value of the analysis of the most relevant parameters. The DTI curves S-wave (peak of the systolic velocity) and the E-wave (peak of early diastolic velocity) of the DTI curves and the peak velocity pf isovolumic contraction (IVC) and relaxation time (IVR) measured on DTI curves. Tissue tracking correspond to the peak systolic displacement

|  | DTI ‘S-wave’  (cm/s) | DTI ‘E-wave’  (cm/s) | DTI ‘A-wave’  (cm/s) | IVC peak velocity  (cm/s) | IVR peak velocity (cm/s) | Peak VTI  (cm) |
| --- | --- | --- | --- | --- | --- | --- |
| Intra-observer absolute variability | 0.04 | 0.06 | 0.12 | 0.01 | 0.23 | 0.23 |
| Intra-observer relative variability | 1% | 1% | 2% | 1% | 18% | 3% |
| Inter-observer absolute variability | 0.24 | 0.14 | 0.27 | 0.34 | 0.23 | 0.13 |
| Intra-observer relative variability | 5% | 3% | 4% | 15% | 23% | 18% |

**Table 3:**

Inter and intra relative and absolute variability for most DTI parameters studied; about 10 patients.(DTI: Doppler tissue imaging; IVC: isovolumic contraction; IVR: isovolumic relaxation : VTI: velocity time integral called also ‘tissue tracking’ peak systolic displacement).
